# Developing Fused Deposition Modeling Additive Manufacturing Processing Strategies for Aluminum Alloy 7075: Sample Preparation and Metallographic Characterization

**DOI:** 10.3390/ma15041340

**Published:** 2022-02-11

**Authors:** Huan Ding, Congyuan Zeng, Jonathan Raush, Kasra Momeni, Shengmin Guo

**Affiliations:** 1 Department of Mechanical and Industrial Engineering, Louisiana State University, Baton Rouge, LA 70803, USA; hding3@lsu.edu (H.D.); czeng8@lsu.edu (C.Z.); 2 Department of Mechanical Engineering, The University of Louisiana at Lafayette, Lafayette, LA 70503, USA; jonathan.raush@louisiana.edu; 3 Department of Mechanical Engineering, The University of Alabama, Tuscaloosa, AL 35487, USA; kmomeni@ua.edu

**Keywords:** aluminum alloy 7075, powder metallurgy, fused deposition modeling, wet-mixing, sintering

## Abstract

Currently, no commercial aluminum 7000 series filaments are available for making aluminum parts using fused deposition modeling (FDM)-based additive manufacturing (AM). The key technical challenge associated with the FDM of aluminum alloy parts is consolidating the loosely packed alloy powders in the brown-body, separated by thin layers of surface oxides and polymer binders, into a dense structure. Classical pressing and sintering-based powder metallurgy (P/M) technologies are employed in this study to assist the development of FDM processing strategies for making strong Al7075 AM parts. Relevant FDM processing strategies, including green-body/brown-body formation and the sintering processes, are examined. The microstructures of the P/M-prepared, FDM-like Al7075 specimens are analyzed and compared with commercially available FDM 17-4 steel specimens. We explored the polymer removal and sintering strategies to minimize the pores of FDM-Al7075-sintered parts. Furthermore, the mechanisms that govern the sintering process are discussed.

## 1. Introduction

Due to their excellent mechanical properties, such as high strength [1], low density [2], and high stress-corrosion-cracking resistance [3], 7xxx series aluminum alloys are commonly employed by the aerospace industry. The widely accepted strengthening mechanism for the 7xxx series aluminum alloy is precipitation strengthening, which is described by the supersaturated solid solution precipitation theory, including Guinier–Preston zone—metastable η′—stable η (MgZn_2_ phase) [4]. To further improve the mechanical properties of 7xxx series aluminum alloys, many researchers focus on particle additions with Al_2_O_3_ [5], SiC [6], B_4_C [7], and high-entropy alloy [8] powders.

Additive manufacturing (AM) techniques provide a unique capability for making components with complex geometries. Indeed, many industrial components, such as turbocharger compressor blades, grippers, and gearboxes, have complex shapes. Several AM technologies have been applied to prepare Al alloy parts, such as laser powder bed fusion (LPBF) AM [9] and electron beam powder bed fusion AM [10]. As an AM process, fused deposition modeling (FDM) has gained much attention in recent years for manufacturing customized polymer-based components rapidly. Polymeric materials such as acrylonitrile butadiene styrene (ABS) [11], poly lactic acid (PLA) [12], and wax [13] are the most popular materials used by FDM. Parts fabricated with these materials can be used for design verification, to create patterns for casting processes, and for medical applications. Researchers are also exploring various metal/polymer filaments to make composites containing metallic particles, such as iron/nylon [14], copper/ABS [15], and Al/Al_2_O_3_/nylon composites [16]. FDM has also been explored to prepare pure metallic AM parts. To fabricate pure metallic AM parts, the FDM printer first prints a green-body using filaments containing metal powders and polymer binders. Then, debinding and sintering are conducted on the printed green-body to form fully dense metallic parts. Typically, the sizes of metallic powders are a few microns in diameter, and the total metal volumetric content in the filament is around 50% or less [17]. The FDM-based metallic part AM technology, also referred to as bound metal deposition (BMD), has two main advantages, i.e., (i) the FDM equipment is low-cost and portable, as the operating temperature to make green bodies is about 190–250 °C [11,12,15], eliminating the need for expensive, high-energy heat sources such as a laser; and (ii) the printing materials are intrinsically safe because filament (powders bonded by polymer binder) is less likely to cause safety concerns than powder-bed-based AM materials.

Despite the advantages, manufacturing aluminum alloy parts using the FDM technique faces the following challenges: first, aluminum alloy powders are covered with a stable aluminum oxide film, which reduces sinterability. Second, aluminum alloy powders in the green-body of FDM parts are loosely packed due to the high concentration of polymeric binder and low extrusion pressure. Third, preparing new FDM filaments with a high concentration of metal powders is challenging. Currently, no FDM filament is commercially available for printing aluminum 7075 (Al7075) parts. Thus, it is necessary to investigate the sintering behavior of Al7075 powder mixed with polymer binder in loosely packed conditions to assess the feasibility of making Al7075 parts using FDM-based AM. Conventional pressing-and-sintering powder metallurgy (P/M) aluminum alloys are usually carried out at high pressing pressures before sintering [8,18,19,20]. For reference [8], the pressing pressure is about 400 MPa, and for reference [20], 250–770 MPa pressure was applied in the compression process. Even for the 250 MPa pressing pressure, the density of the green-body reached about 92%. To study the loosely packed specimen sintering, a low pressing pressure is used in this study.

**Figure 1:**
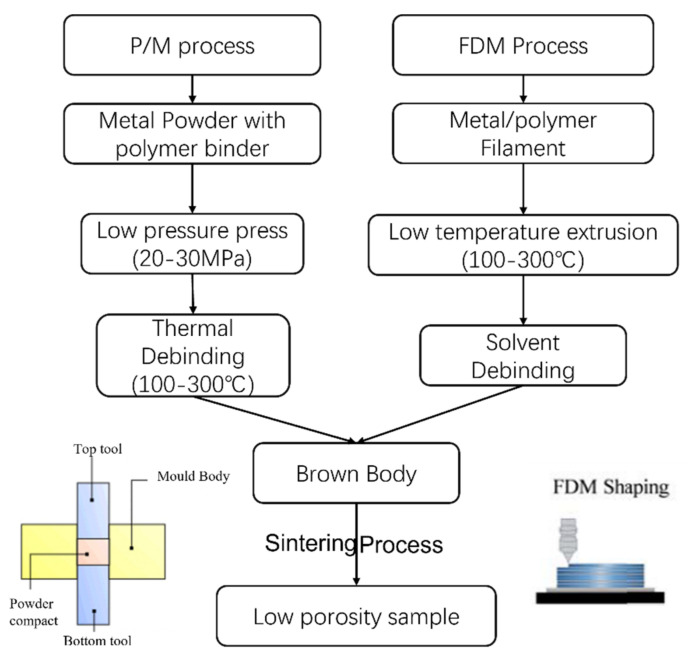
P/M-based specimen preparation method for the study of metallic FDM AM processing strategies.

Since the FDM specimen is in a loosely packed state, robust aluminum alloy parts can be made with the FDM method only with optimized processing control. In this paper, considering the metal-based FDM process shares many commonalities with the classical pressing and sintering-based P/M process—including raw materials mixing, extrusion/consolidation, and controlled sintering (Figure 1)—the classical pressing and sintering-based P/M method is explored to investigate the feasibility of making Al7075 parts using the FDM process. To mimic the FDM processed green-bodies, which contain high levels of metallic powders and polymer binders, Al7075 powders and polymer binders are first mixed and consolidated with low pressure. We utilized wet-mixing of polymer binder with Al7075 powders to achieve good mixing. In the powder injection molding (PIM) industry, the mixed polymer consists of backbone, filler, and lubricant. Then, thermal debinding is performed to remove a portion of the binder before the sintering step. Alloys suitable for producing high-quality metallic FDM AM parts (not limited to Al7075) and the relevant processing windows can be rapidly screened with the P/M-based specimen preparation method presented in this paper.

## 2. Materials and Methods

**Figure 2:**
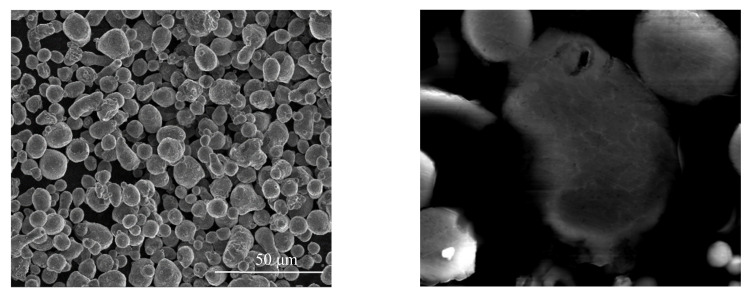
Images showing the shape and size of the raw Al7075 powders.

**Figure 3:**
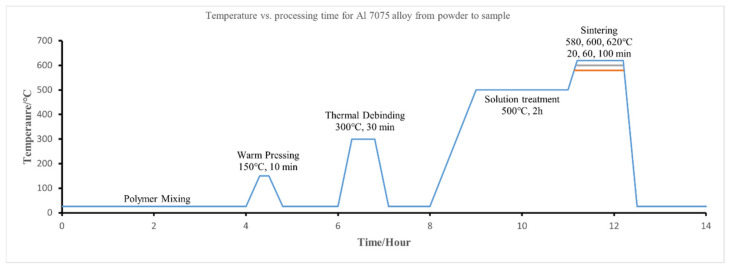
The typical temperature profile for making FDM-like bulk Al7075 specimens with the classical pressing and sintering-based P/M method.

Al7075 powders (supplied by American Elements, Pasadena, CA, USA, Table 1), polyethylene oxide (PEO) powders, and polypropylene (PP) powders were used in this study. Figure 2 shows that the original Al7075 powders were close to a spherical shape with an average diameter of 9 μm. Figure 3 shows the temperature profile for making FDM-like Al7075 bulk specimens from the raw materials. The polymer consisted of PP, PEO, and stearic acid (SA) in a mass ratio of 6:3:1, which is 60% backbone (PP), 30% filler (PEO), and 10% lubricant (SA). The mass ratio of the total polymer to Al7075 powder was 1:12. The polymer was dissolved in an appropriate amount of toluene before the Al7075 powders were added to achieve good mixing. To ensure that the polymer was thoroughly dispersed in toluene, the mixture was heated to 100 °C. After entirely dissolving the polymer binder in toluene, the Al7075 powder was added and dispersed using an ultrasonic shaker for 5 min. Then, 30 min of electromagnetic stirring was carried out. Finally, after evaporating all the solvent at room temperature (36 h), Al7075 powders coated with polymer binder were collected as the precipitates. A sieve with 75 μm mesh size was applied to the precipitates to eliminate large agglomerates. Using the Al7075/polymer powder mixture, disk-shaped specimens with a diameter of 12.7 mm and a thickness of 3 mm were prepared in a warm-compression process with a temperature and pressure of 150 °C and 20 MPa, respectively. The pressing step was conducted in air. A debinding process was implemented at 300 °C for 30 min, also in air.

To better understand the sintering process, the phase diagram and liquid phase content for the Al7075 alloy at different temperatures were calculated using Thermo-Calc software with database TCAL6. The typical sintering process consists of two stages to obtain densified specimens: stage 1 is to heat the specimen to 500 °C for two hours with a 10 °C/min heating ramp rate. Stage 2 is to further heat the specimen to 580 °C, 600 °C, or 620 °C for 20, 60, or 100 min, with a heating rate such as 10 °C/min (as shown in Figure 3). Different sintering durations, heating rates, and gas environments were also explored to improve the sintering process. All the sintering processes were performed in either a custom vacuum furnace or a Centorr Series LF top loading multipurpose vacuum furnace. The relative density and porosity of the specimens were measured by the Archimedean method. A scanning electron microscope (SEM) was utilized to investigate the distribution of the polymers in the matrix and the sample microstructures. X-ray diffraction (XRD) tests were conducted with a Panalytical Empyrean multipurpose diffractometer, equipped with Pre-FIX modules with Cu Kα radiation. Microhardness tests were performed on the sintered specimens using a CM-802 AT microhardness tester, with a test load of 500 gf and a dwell time of 15 s.

## 3. Results and Discussion

### 3.1. Forming FDM-Like Al7075/Polymer Binder Mixture

**Figure 4:**
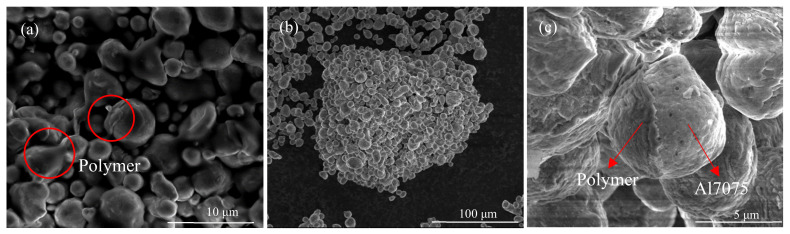
SEM images showing the morphology of the powder/binder mixture. (**a**) Commercial 17-4 steel filament. (**b**,**c**) Wet-mixing processed Al7075 powder with the polymer binder.

FDM utilizes a filament containing metal powder/polymer binder mixture to prepare metallic AM parts. Figure 4a shows the morphology of a commercial FDM 17-4 stainless steel filament, in which the polymer binder partially coats the metal powder particles. Figure 4b,c show the morphologies of the Al7075 powder/polymer binder mixture prepared using the proposed wet mixing processes. After the wet mixing process, the polymer binder covers the surface of the Al7075 powder evenly. Spherical-shaped metallic powder particles usually would not form mechanical interlocking under relatively low compressing forces at low temperatures. For Al7075 particles covered with a polymer coating, the polymer binder provides weak bonding between metal particles, which is why clusters of powder are observed in the wet-mixed powder/binder mixture. In contrast, no such clusters can be found in the raw Al7075 powder (Figure 2). For both cases, the polymer binder is bonded to the metal particles in a similar way, as indicated by the red arrows and circles in Figure 4, proving the proposed wet-mixing method can effectively produce an FDM-like Al7075/polymer binder mixture.

### 3.2. Forming FDM-Like Bulk Al7075 Samples

During the FDM fabrication process, the printer heats the filament to soften the polymer binder and then extrudes the filament to form the green-body via layer-by-layer deposition. In this study, a compression process was applied to the powder/polymer mixture to mimic the green-body formed in the FDM process. The main criterion for selecting the compression process parameters was to avoid any plastic deformation of the Al7075 particles. The yield strength and hardness of an alloy can be related based on Tabor’s equation $\sigma_{y}=\left(\frac{HV}{3}\right)-182.3$, where $\sigma_{y}$ is the yield stress and HV is the microhardness. According to references [21,22], the Vickers hardness of the air-atomized Al7075 powder is about 200 HV. Therefore, the yield strength of the Al7075 powder was estimated to be around 472 MPa. In the compression process, a small pressure of 20 MPa was applied, together with a temperature of 150 °C. The 150 °C pressing temperature was chosen to mimic the typical FDM extrusion temperature (about 100–300 °C) and to ensure a similar polymer behavior. The contact pressure may be significantly greater than the pressing pressure for powder packs. As the applied pressing pressure of 20 MPa is much lower than the yield strength, no visible plastic deformation was observed on the compressed Al7075 particles.

**Figure 5:**
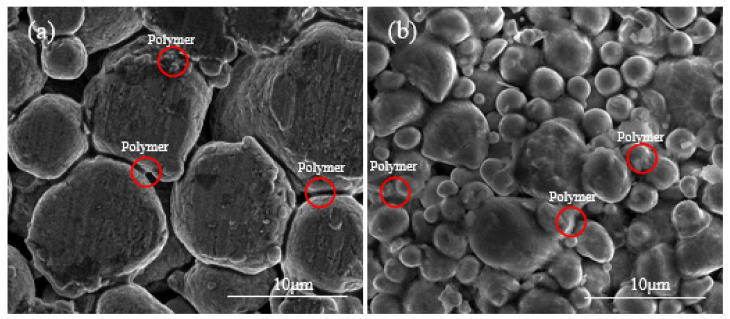
SEM images display the morphology of the specimens. (**a**) Compressed Al7075 sample. (**b**) Washed FDM 17-4 steel sample.

Figure 5 shows the SEM images for both the warm-pressed Al7075 powder/polymer binder specimen and the 17-4 steel specimen prepared by a commercial FDM process. For the 17-4 steel specimen, a chemical washing step using the Markforged Wash-1 system was applied to form the so-called brown-body from the green-body. According to Figure 5b, in the FDM 17-4 steel brown-body, polymer binder was uniformly dispersed between metal powders. Likewise, the polymer was reasonably uniformly distributed between the Al7075 particles in the Al7075 powder/polymer binder bulk sample (Figure 5a). Due to the low compression force used, the Al7075 particles were pushed together without any plastic deformation, as seen in Figure 5a. In addition, the low compression force is not expected to break down the oxide film on the surfaces of the Al7075 particles, just as in the FDM extrusion process. The FDM-like bulk Al7075 samples were successfully formed using the proposed compression process in this study.

### 3.3. Forming FDM-Like Al7075 Brown-Body via Thermal Debinding

Filaments containing metallic particles and polymer binders are used to first make the green-body to produce metallic parts with the FDM process. Subsequently, a portion of the polymer binder must be removed via either chemical washing or thermal debinding to form the so-called brown-body prior to the sintering stage. For example, the debinding process of the commercial FDM 17-4 specimen is divided into two steps, in which the chemical washing would first partially remove the polymer. Then, the thermal debinding would remove the backbone polymer. To better simulate the debinding process of FDM samples, a two-step thermal debinding process was adapted. This study used a polymer mixture of PP, PEO, and SA. The process was designed such that the PEO (with a boiling point of about 250 °C) was to be removed in a first thermal debinding process at 300 °C to develop the Al7075 brown-bodies, and PP and SA were removed during the second thermal debinding stage (500 °C for two hours).

To determine the polymer contents in the green-body and brown-body of both P/M compressed specimens and commercial FDM printed specimens, the weight loss method was used, and the results are displayed in Table 2. The total weight loss of the 17-4 stainless steel specimens prepared by commercial FDM and Al7075 samples prepared by P/M compression was about 9.0 ± 0.6% and 5.1 ± 0.4%, respectively. According to the original mass ratio between the polymer and Al7075 powder (1:12), the polymer content should be 7.7%. The decrease in mass fraction of polymer in the Al7075/polymer specimen was caused by the removal of Al7075/polymer agglomerates larger than 75 μm during the particle selection process. The commercial FDM 17-4 stainless steel filament polymer content was around 9–10%.

**Figure 6:**
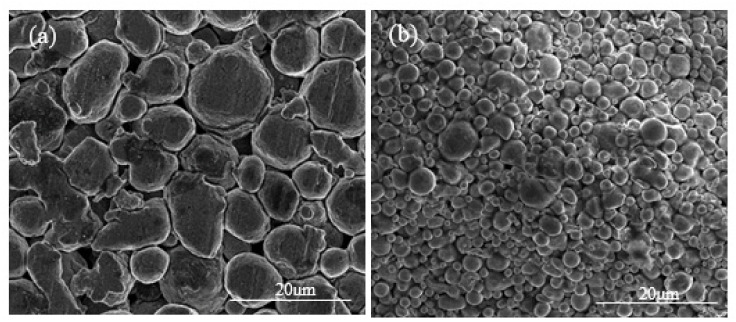
The microstructure of the specimens after debinding. (**a**) Al7075 with wet-mixing powders (two-step thermal debinding). (**b**) FDM 17-4 steel specimen (chemical washing and thermal debinding).

Comparing the microstructures of the bulk Al7075 alloy samples prepared in this study and the commercial FDM-prepared 17-4 steel samples, no apparent presence of polymer was observed in the Al7075 specimens after thermal debinding (Figure 6). Similarly, only minute amounts of polymers remained in the commercial FDM 17-4 stainless steel samples after debinding.

### 3.4. Sintering Strategies for FDM-Like Al7075 Samples

The bulk Al7075 alloy samples displayed the typical FDM-like morphology with loosely packed metal particles. Due to the large gaps between the Al7075 particles and the presence of surface oxidation, it is challenging to form high strength/low porosity Al7075 parts in the sintering process. Different sintering strategies were explored with the suitable powder morphology to form strong FDM Al7075 parts.

Densification during the sintering of aluminum alloys typically has the following mechanisms [23]:

1. Solid-state sintering is controlled mainly by atomic diffusions, such as surface diffusion, grain-boundary diffusion, and volume diffusion;
2. Particle rearrangement by capillary forces;
3. Solution and reprecipitation, with particle dissolution in the liquid phase and subsequent reprecipitation at sites of lower chemical potential; and
4. Pore filling, where the liquid phase will fill the pores during the sintering process.

**Figure 7:**
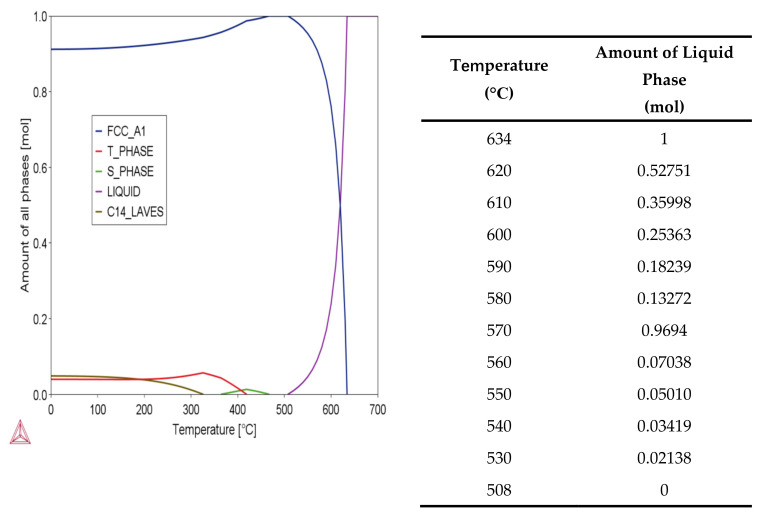
Phase diagram and the amount of liquid phase at different temperatures.

Pore filling is an essential densification mechanism in the sintering of aluminum alloys [24] for which liquid phase sintering (LPS) is necessary. The amount of liquid phase in a typical LPS system is 5–30% [25]. We utilized Thermo-Calc software to calculate the phase diagram, Figure 7, to identify the amount of liquid phase present at different temperatures for the Al7075 alloy. Considering the presence of a large number of pores in the loosely packed specimens, 580 °C (13 vol % liquid phase), 600 °C (25 vol % liquid phase), and 620 °C (53 vol % liquid phase) were adapted as the sintering temperatures to test the sintering behavior of the Al7075 specimens, as seen in Table 3.

**Figure 8:**
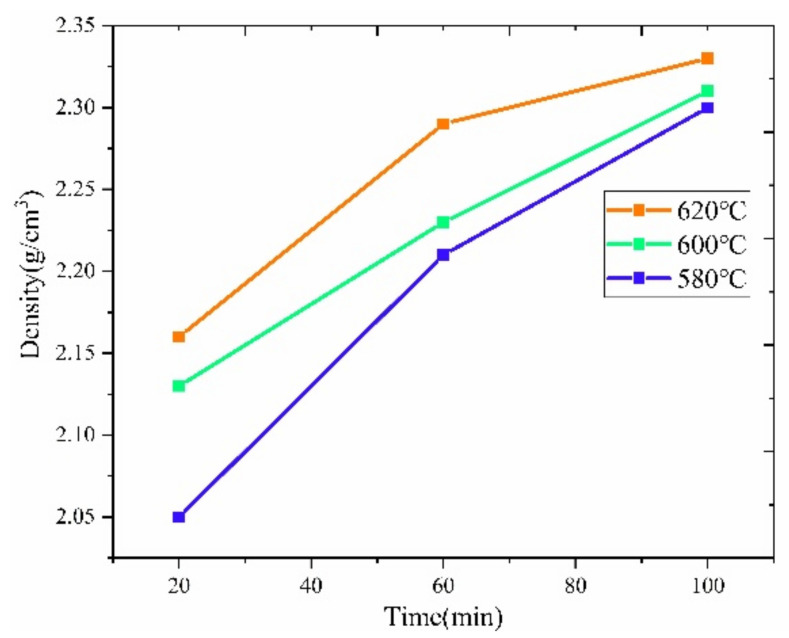
The density of the Al7075 specimen sintered under vacuum.

The first set of sintering tests under the so-called standard sintering strategy was conducted under vacuum. Figure 8 illustrates the variation of density achieved for the sintered specimens with the range of sintering temperature. The density of the specimens improves by increasing either the sintering temperature or the sintering time. In the first stage of the study, the highest specimen density was 2.33 g/cm^3^ (620 °C, 100 min). Considering that the theoretical density of Al7075 is 2.81 g/cm^3^, the highest relative density was around 83%, indicating that even after 100 min of sintering at 620 °C with theoretical 53 vol % liquid content, the FDM Al7075 specimen remained in the early stages of sintering.

**Figure 9:**
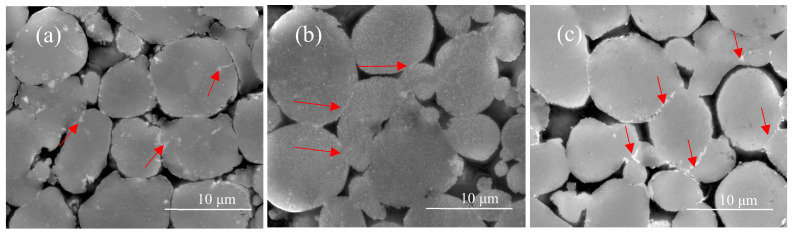
Microstructures of the specimens sintered at different temperatures and times in the vacuum. (**a**) 580 °C, 100 min. (**b**) 600 °C, 100 min. (**c**) 620 °C, 100 min.

Figure 9 shows the SEM images of Al7075 specimens sintered under vacuum, with many pores remaining in the as-sintered specimens. In aluminum alloy sintering, the liquid phase usually has a light color under SEM and is distributed along the particle boundaries [26,27]. Figure 9a shows that the solidified liquid is distributed at the interface or inside Al7075 particles, observed as the light or white phase without any fixed pattern. As the sintering temperature increases, the liquid phase gradually accumulates towards the particle contact regions or particle boundaries. Aggregation of the white liquid phase was observed in the sintered neck regions. In addition, the sintering reaction seems to occur only in the regions with inter-particle contacts. This means that the contact flattening sintering mechanism [28] dominates the sintering process under the current sintering conditions. The contact flattening mechanism is usually caused by a pressure imbalance between the liquid and external pressure (ambient pressure in the sintering chamber) during the sintering process, following the equation:

$$P_{l}=P_{out}-\frac{2\gamma_{lv}}{r}$$

where the *P_out_* is the external pressure, $\gamma_{lv}$ is the liquid/vapor interfacial energy, and *r* is the radius of the liquid meniscus. The pressure in the liquid is always lower than the external pressure. Therefore, the particles are under compression, which promotes the formation of a sintering neck. As the sintering temperature increases from 580 °C to 620 °C (liquid phase fraction increases), the amount of liquid phase in the contact area increases (Figure 9c) and the atomic diffusion process is enhanced, which further promotes the growth of the sintering neck.

**Figure 10:**
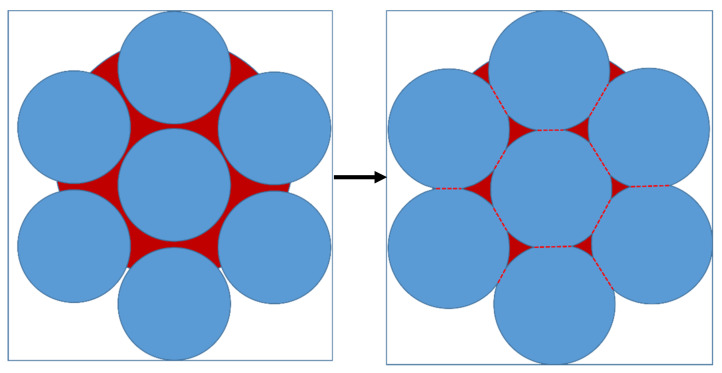
The evolution of liquid phase sintering.

**Figure 11:**
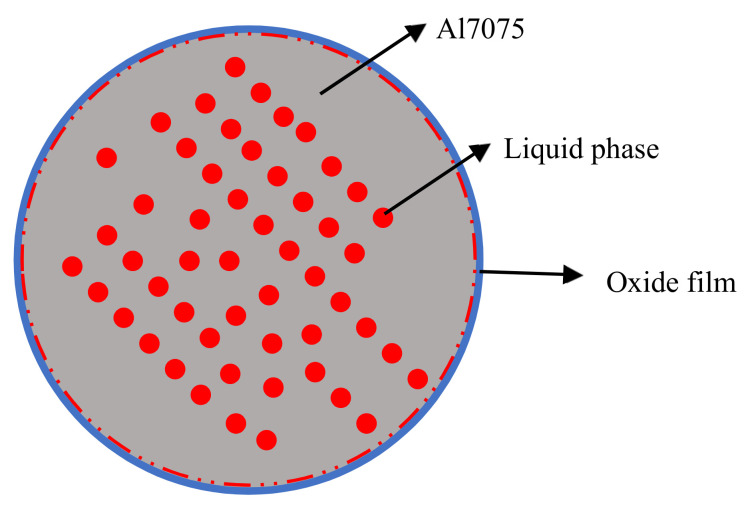
Liquid phase during the Al7075 pre-alloyed powder sintering process.

In the Al7075 alloy system, the generated liquid phase may be related to Mg, Cu, and Zn elements. Based on the phase diagrams calculated by the Thermo-Calc software package, the content of Al, Mg, Cu, and Zn in the liquid phase was determined. For example, when the sintering temperature is 620 °C, the mass fraction of Al, Mg, Cu, and Zn in the liquid was 85.7%, 3.8%, 2.7%, and 7.8%, respectively. In theory, under the current sintering conditions, especially at 620 °C, a large amount of liquid phase would be generated between the particles. The particles would undergo rearrangement and dissolution-precipitation by capillary forces. Usually, the diffusion of atoms in the liquid phase is faster in mass transfer, ensuring rapid densification, as seen in Figure 10. In addition, a high volume of liquid phase would promote the distortion of the parts during the sintering. However, none of the above sintering phenomena were observed in the first batch of sintering tests. One of the reasons is that an Al_2_O_3_ layer covers the aluminum powder surfaces due to the high affinity between aluminum and oxygen, which hinders the sintering process by reducing wettability and diffusivity [29]. The oxide is thermodynamically very stable, and the liquid phase produced during the sintering process is encapsulated inside the single particles, making it difficult to penetrate the oxide film, as seen in Figure 11. In this study, atomized Al7075 powder is used, and all powder particles have the same evenly distributed composition.

The liquid phase primarily forms within particles upon heating to a temperature between solidus and liquidus temperatures. The sintering mechanism of Al7075 atomized powder is a super solidus sintering. The liquid phase appears spontaneously in the particle contact area and within the powder particle. Due to the presence of the oxide film on the surface of the pre-alloyed powder, the liquid phase produced during the sintering process is covered by the oxide film, as seen in Figure 11, and it will prevent sintering in the non-contact areas of the particles. Only in the area of inter-particle contact is the formation of the sintering neck promoted, which is consistent with the experimental results. The average sintering neck size of the 580 °C, 600 °C, and 620 °C sintered specimens is 1.8 ± 0.6 µm, 2.2 ± 0.7 µm, and 4.4 ± 1.2 µm, respectively.

**Figure 12:**
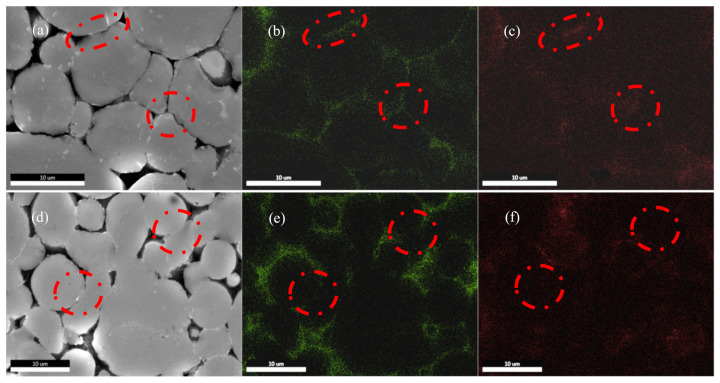
EDS Mapping of sintered specimens. (**a**) 580 °C, 100 min. (**b**) O. (**c**) Mg. (**d**) 620 °C, 100 min. (**e**) O. (**f**) Mg.

EDS mapping was adopted to further investigate the elemental diffusion behavior in the sintered specimens. Figure 12 depicts the EDS mapping of the sintered specimen. For the low sintering temperature (580 °C), it can be observed that oxygen (O) elements aggregate at the grain boundaries. In addition, the net structure formed by the aggregation of O elements is almost continuous, indicating that the liquid phase hardly penetrates the oxide film during the sintering process to promote the diffusion process. For the high sintering temperature (620 °C), a break in the net structure oxide film is observed, corresponding to the sintered necks’ location. It shows that increasing the volume fraction of the liquid phase has a positive effect on sintering. Magnesium (Mg) is reported to diffuse to the aluminum–aluminum oxide interface and to react with Al_2_O_3_ to form a spinel during the sintering process with the following reaction equation [30]:

3Mg + 4Al_2_O_3_ → 3MgAl_2_O_4_ + 2Al

This reaction can rupture the oxide film and expose the underlying sintering liquid phase. When contact areas exist between the particles, the liquid phase in the contact area promotes the diffusion of the elements. It enhances the dissolution and precipitation mechanisms, which contributes to the growth of the sinter neck. From the magnesium EDS mapping of the 580 °C specimen, there is a tendency for magnesium to accumulate in the particle contact regions. However, the appearance of sintered necks is not observed in the contact area. This is because the liquid phase volume fraction is small when the sintering temperature is 580 °C. The liquid phase within each particle cannot wet and break the oxide film in the available time. For the 620 °C specimen, as the amount of liquid phase rises, the oxide film in the particle contact region is broken, and a sintering neck is formed. Although magnesium can break the oxide film of the non-contact region of the particles, the element is unable to diffuse through the pores, which leads to the aggregation of the Mg element in the non-contact regions.

**Figure 13:**
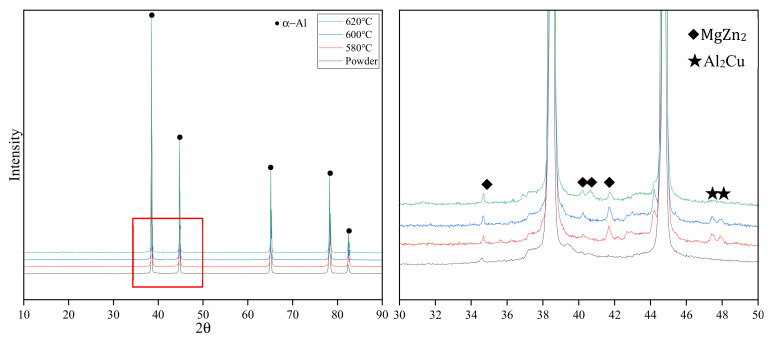
XRD of sintered specimens with a sintering time of 100 min.

Figure 13 shows the XRD patterns of sintered Al7075 alloy specimens (where sintering time was 100 min). The major secondary phases in all the sintered specimens are MgZn_2_ and Al_2_Cu. Reference [31] pointed out that the formation enthalpy (ΔH) decreases in the order MgZn_2_ > Al_2_Cu, whereas the binding energy (E_b_) decreases in the order Al_2_Cu > MgZn_2_. The formation of MgZn_2_ can be promoted by increasing the content of Zinc or decreasing the content of magnesium. In a low liquid phase fraction sintering process (580 °C), even in the particle contact area, it is difficult for the liquid phase inside the particles to wet the oxide film and promote the magnesium and Al_2_O_3_ layer reaction. The content of magnesium in the matrix remains almost unchanged. In a high liquid phase fraction sintering process (620 °C), the reaction of the magnesium with the oxide film consumes the magnesium in the matrix, which drives the formation of MgZn_2_. In the 620 °C specimen XRD patterns, Al_2_Cu peaks disappeared, and only MgZn_2_ was detected, which is consistent with the results obtained from the theoretical model.

Table 4 shows the microhardness of sintered Al7075 alloys. According to the literature [32], the strengthening mechanism of sintered aluminum specimens is mainly secondary hard phase dispersion strengthening. With the increase of sintering temperature and sintering time, the microhardness of the specimens showed an increasing trend. Based on the XRD results, more MgZn_2_ reinforced phases are present in the 620 °C specimens. Additionally, microhardness is associated with the relative density since the specimens were not fully dense. As the sintering temperature increased, the amount of liquid phase in the particles increased. The increased amount of liquid phase further promoted the formation of the sintering neck and enhanced inter-particle connectivity. As a result, the specimen with a sintering temperature of 620 °C and a sintering time of 100 min showed the highest microhardness of 74.1 HV due to the highest density.

Table 5 shows the microhardness of Al7075 alloys with different manufacturing methods. It can be found that the microhardness of the P/M specimen (sintered at 620 °C for 100 min) was almost equal to the forged Al7075, even under non-densified sintering conditions. Further, all specimens with different manufacturing technologies [33,34,35] have undergone careful post-heat treatments. From this aspect, the as-fabricated P/M specimen displayed excellent microhardness properties.

The primary problem with the standard sintering strategy is that loosely packed specimens do not generate sufficient contact areas to accelerate the diffusion of atoms during the sintering process. The formation and growth of the sintered neck is very slow. Further, the generated liquid phase makes it hard to completely wet and penetrate the oxide film on the particle surface. The pore-filling mechanism cannot work under the standard sintering strategy since the loosely packed specimens have large pore sizes. Therefore, the sintered specimens remain at the early stage of sintering and are limited by sintering neck growth.

**Figure 14:**
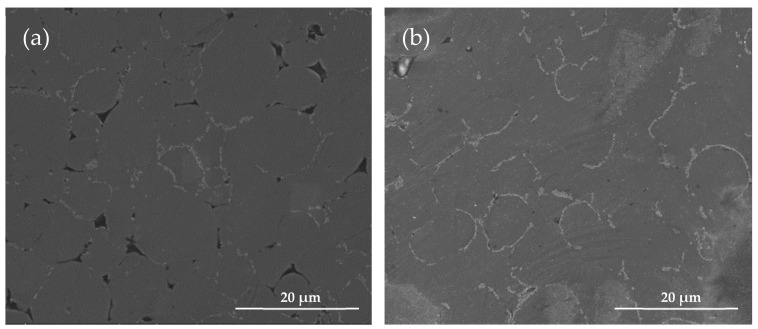
Specimen sintered at 620 °C for (**a**) 4 h and (**b**) 8 h.

For the specimen sintered at 620 °C for 100 min, it can be observed that the size of the sintered neck increases significantly. At the same time, still, no pore filling is observed in the sintered specimen. The more liquid phase generated in the inter-particle area during sintering will contribute to the breakage of the oxide film and the formation of a sinter neck. Once the sintering neck is formed, the growth of the sintered neck is controlled by the diffusion of atoms and the solution and reprecipitation mechanisms. The sintering rate theoretically converges to the solid-phase sintering rate. Although the solid-phase sintering rate is slow, densification of the specimen can be achieved by extending the sintering time. Figure 14 shows the SEM images for the specimens sintered at 620 °C for 4 h and 8 h. As the sintering time increases, the porosity gradually decreases, and the white liquid phase marks appear around the pores. This indicates that as the sintering time increases, the atomic diffusion is promoted, and thus the sintering neck grows sufficiently, and, finally, the specimen reaches full densification at a sintering time of 8h. Then, the liquid phase in the specimen forms a distributed network-like shape in the matrix.

**Figure 15:**
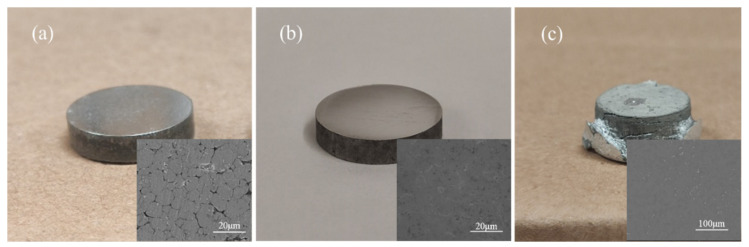
Sintered Al7075 specimens with different heating rates to 580 °C for 20 min. (**a**) 10 °C/min. (**b**) 30 °C/min. (**c**) 60 °C/min. The inserted SEM images indicate the microstructures of the corresponding sintered specimens.

Some researchers have pointed out that increasing the heating rate of the sintering temperature can increase the difference in thermal expansion during aluminum sintering [36]. Due to the high porosity and low contact area, the rate of the densification process of the specimen with the standard sintering strategy is very slow. Using the difference in powder expansion to modify the inter-particle contact area to speed up the densification process becomes a feasible method. Although thermal expansion is complex, a high heating rate is usually associated with a large temperature gradient. However, considering that the specimen is in a loosely packed state, a heating rate that is too fast can lead to localized excessive expansion and solid–liquid imbalance, which can damage the structure of the specimen. Therefore, a suitable heating rate needs to be found. Figure 15 shows the sintered specimen with different heating rates. When the heating rate increases to 30 °C/min, the porosity in the specimen decreases significantly even when the sintering temperature is 580 °C. Increasing the heating rate induces the specimen to produce more sintering neck in a shorter period. When the heating rate increases to 60 °C/min, the excessive thermal expansion imbalance destroys the specimen structure. Although full densification is achieved in some areas of the specimen, the specimen as a whole has failed.

The gas environment has an important influence on the sintering of aluminum alloys. Nitrogen is widely regarded as necessary [24]. It is generally believed that aluminum reacts with nitrogen to form AlN, which will reduce the pressure in the closed pores relative to the external pressure, and then promote pore filling. To keep the specimen compressed by the external atmosphere during the sintering process, after pulling vacuum, argon gas at 1 bar is refilled during the late sintering process. Once the sintering process starts, sintered necks form in the inter-particle contact areas, which leads to closed pores. If the gas pressure in the closed pores is lower than the external pressure, locally, the specimen will be in a compression state. The liquid phase generated in the particle contact region transmits the resulting compressive forces, making the chemical potential of the atoms in the contact region higher than that of the atoms in the non-contact region. The result is the dissolution of the particles in the contact area, formation of the sinter neck, and facilitation of the diffusion process of the atoms.

**Figure 16:**
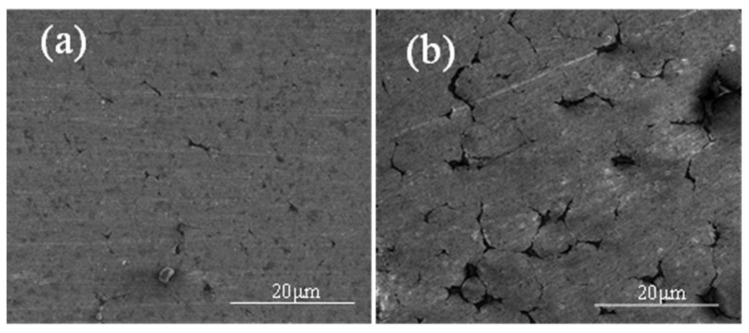
SEM images showing the sintered Al7075 specimens under different sintering atmospheres to 580 °C for 20 min with applied (**a**) argon and (**b**) vacuum.

Figure 16 shows the sintered specimens with different sintering atmospheres. As mentioned above, more sintering neck forms in the inter-particle contact area when the external gas pressure is increased. Table 6 shows the density and hardness of the Al7075 specimens for the optimized sintering strategies. Compared to the standard sintering strategy, the optimized sintering parameters greatly improve the sintering quality.

The density of the FDM Al7075 specimens can be significantly improved by increasing the sintering time, heating rate, and applying an argon environment during the sintering process. Subsequent testing of the mechanical properties of the specimens prepared under the optimized sintering strategies will be reported in the future.

## 4. Conclusions

This study uses a classical pressing and sintering-based P/M method to explore the feasibility of utilizing Al7075 in a fused deposition modeling AM process. A systematic experimental investigation has been conducted, including the Al7075 powder/polymer binder mixture preparation, bulk sample fabrication, thermal debinding, and sintering process optimization. The following conclusions can be reached:

(1) Employing the wet-mixing method, it is feasible to prepare Al7075/polymer composites which mimic the FDM-processed green-body/brown-body. The microstructures of the Al7075 specimens at all P/M stages are similar to those of the commercially available 17-4 steel FDM specimens.
(2) The sintering mechanism of the pre-alloyed Al7075 powder is super solidus sintering. The liquid phase comprises Al, Zn, Mg, and Cu. The slow heating rate of 10 °C/min hinders the Al7075 sintering densification process due to the high porosity and low particle contact area.
(3) By optimizing the sintering parameters, such as increasing the sintering heating rate to 30 °C/min and providing an argon pressure after the pores are closed, the density of the sintered Al7075 specimens can be significantly improved. The highest density and microhardness achieved were 2.72 g/cm^3^ and 98.4 HV, respectively.
(4) It is feasible to prepare robust Al7075 parts using the FDM-based AM process, provided the right filament preparation, thermal debinding, and sintering parameters are taken.

## Figures and Tables

**Table 1:** Chemical composition (wt %) of Al7075 alloy.

		Zn	Mg	Cu	Fe	Si	Cr	Ti	Mn	Ca	Ni
Powder	Min	5.1	2.1	1.2	0	0	0.18	0	0	0	0
	Max	6.1	2.9	2.0	0.50	0.40	0.28	0.20	0.30	0.03	0.03

**Table 2:** Weight loss after debinding and sintering.

	Original Specimen (g)	Sintered Specimen (g)	Total Weight Loss after Sintering	Wash/Thermal Debinding Mass Loss
FDM 17-4 (FDM printed)	8.19	7.40	9.7%	4.5%
	8.20	7.50	8.5%	4.1%
	7.90	7.20	8.9%	4.2%
Al7075/polymer (Powder after selection)	1.52	1.44	5.1%	3.2%
	3.46	3.29	5.0%	3.0%
	5.43	5.13	5.5%	3.4%
Al7075/polymer (Compressed specimen)	1.33	1.27	4.5%	2.7%
	6.82	6.47	5.2%	3.5%

**Table 3:** Different sintering strategies for FDM-like Al7075 alloy brown-bodies.

	Sintering Temperature (°C)	Sintering Time (min)	Heating Rate (°C/min)	Sintering Atmosphere
Standard sintering strategy	580	20/60/100	10	Vacuum (6.7 × 10^−3^ Pa)
	600			
	620			
Optimized sintering strategy	620	240/480	10	Vacuum (6.7 × 10^−3^ Pa)
	580	20	40	Vacuum (6.7 × 10^−3^ Pa)
	580	20	10	Argon (20 mL/min)

**Table 4:** Microhardness of sintered specimens.

Sintering Temperature (°C)	Sintering Time (Minute)	Microhardness (HV)	Error Bar (Standard Deviation)
580	20	31.9	±3.2
	60	36.2	±1.6
	100	50.5	±1.2
600	20	54.3	±2.3
	60	59.5	±3.4
	100	65.4	±1.3
620	20	56.6	±1.2
	60	60.6	±1.8
	100	74.1	±1.4

**Table 5:** Microhardness of Al7075 with different manufacturing technologies.

Type	Microhardness (HV)
LPBF Al7075	94 [33]
Forged Al7075	75 [34]
SPS Al7075	140 [35]
P/M wet-mixing Al7075	74.1

**Table 6:** Density and microhardness of Al7075 with optimized sintering strategies.

Type	Density (g/cm^3^)	Microhardness (HV)
Sintered specimen at 620 °C for 4/8 h	2.66/2.75	58.8/54.3
Sintered specimen with higher heating rate	2.72	98.4
Sintered specimen with argon	2.64	69.6
